# Supplementary material for: Complexome profiling of the Chlamydomonas psb28 mutant reveals TEF5 as an early PSII assembly factor
Source: Plant Cell. 2025 Mar 17;37(6):koaf055. doi: 10.1093/plcell/koaf055 (PMC12164590; doi:10.1093/plcell/koaf055)
Supplement: koaf055_Supplementary_Data [file koaf055_supplementary_data.zip › psb28_interactive.zip!/readMe.pdf]

psb28 complexome profiling

Repositories

- NFDI DataPlant repository: https://git.nfdi4plants.org/venn/psb28_complexomeprofiling_chlamy
- PRIDE Proteome exchange ID: PXD023478

Prepare data set

- Save the psb28 folder to a local directory (do not use cloud storages)
- If necessary unblock plots.zip archive
    - right click -> properties -> unblock
- Extract the archive
- Open “summaryTable/summaryTable.xlsx”

Working with summaryTable.xlsx

- The overview Excel table is interactive. If you extracted the plots folder to the correct relative path and your excel instance allows to open files, you can click on any of the first four columns to open the respective figures.

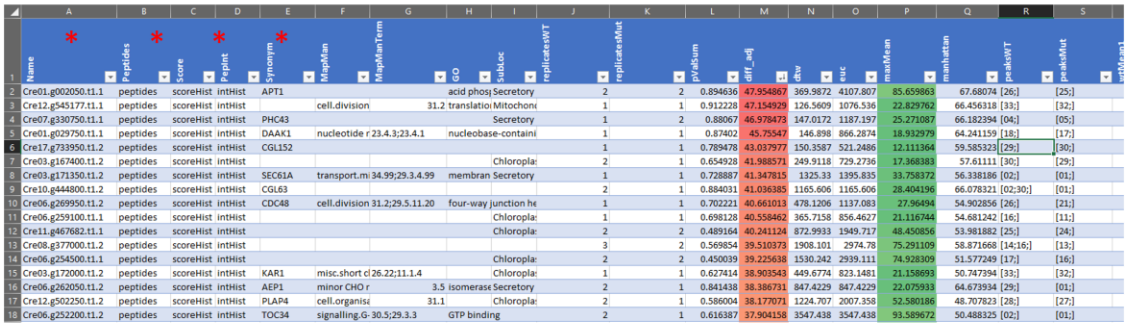

	Name *	Peptides *	Score *	PepInt *	Synonym	MapMan	MapManTerm	GO	SubLoc	replicatesWT	replicatesMut	pValSum	diff_adj	dtw	euc	maxMean	manhattan	peaksWT	peaksMut	wtMean1
2	Cre01.g002050.t1.1	peptides	scoreHist	intHist	APT1			acid phos|	Secretory	2	2	0.894636	47.954867	369.9872	4107.807	85.659863	67.68074	[26;]	[25;]	
3	Cre12.g545177.t1.1	peptides	scoreHist	intHist		cell.division	31.2	translatio	Mitochon	1	1	0.912228	47.154929	126.5609	1076.536	22.829762	66.456318	[33;]	[32;]	
4	Cre07.g330750.t1.1	peptides	scoreHist	intHist	PHC43				Secretory	1	2	0.88067	46.978473	147.0172	1187.197	25.271087	66.182394	[04;]	[05;]	
5	Cre01.g029750.t1.1	peptides	scoreHist	intHist	DAAK1	nucleotide r	23.4.3;23.4.1	nucleobase-containi		1	1	0.87402	45.75547	146.898	866.2874	18.932979	64.241159	[18;]	[17;]	
6	Cre17.g733950.t1.2	peptides	scoreHist	intHist	CGL152					1	1	0.789478	43.037977	150.3587	521.2486	12.111364	59.585323	[29;]	[30;]	
7	Cre03.g167400.t1.2	peptides	scoreHist	intHist					Chloropla:	2	1	0.654928	41.988571	249.9118	729.2736	17.368383	57.61111	[30;]	[29;]	
8	Cre03.g171350.t1.2	peptides	scoreHist	intHist	SEC61A	transport.mi	34.99;29.3.4.99	membran	Secretory	1	1	0.728887	41.347815	1325.33	1395.835	33.758372	56.338186	[02;]	[01;]	
9	Cre10.g444800.t1.2	peptides	scoreHist	intHist	CGL63					2	1	0.884031	41.036385	1165.606	1165.606	28.404196	66.078321	[02;30;]	[01;]	
10	Cre06.g269950.t1.2	peptides	scoreHist	intHist	CDC48	cell.division	31.2;29.5.11.20	four-way junction he		1	1	0.702221	40.661013	478.1206	1137.083	27.96494	54.902856	[26;]	[21;]	
11	Cre06.g259100.t1.1	peptides	scoreHist	intHist					Chloropla:	1	1	0.698128	40.558462	365.7158	856.4627	21.116744	54.681242	[16;]	[11;]	
12	Cre11.g467682.t1.1	peptides	scoreHist	intHist					Chloropla:	2	2	0.489164	40.241124	872.9933	1949.717	48.450856	53.981882	[25;]	[24;]	
13	Cre08.g377000.t1.2	peptides	scoreHist	intHist						3	2	0.569854	39.510373	1908.101	2974.78	75.291109	58.871668	[14;16;]	[13;]	
14	Cre06.g254500.t1.1	peptides	scoreHist	intHist					Chloropla:	2	2	0.450039	39.225638	1530.242	2939.111	74.928309	51.577249	[17;]	[16;]	
15	Cre03.g172000.t1.2	peptides	scoreHist	intHist	KAR1	misc.short c	26.22;11.1.4		Chloropla:	1	1	0.627414	38.903543	449.6774	823.1481	21.158693	50.747394	[33;]	[32;]	
16	Cre06.g262050.t1.2	peptides	scoreHist	intHist	AEP1	minor CHO r	3.5	isomerase	Secretory	2	1	0.841438	38.386731	847.4229	847.4229	22.075933	64.673934	[29;]	[01;]	
17	Cre12.g502250.t1.2	peptides	scoreHist	intHist	PLAP4	cell.organisa	31.1		Chloropla:	2	1	0.586004	38.177071	1224.707	2007.358	52.580186	48.707823	[28;]	[27;]	
18	Cre06.g252200.t1.2	peptides	scoreHist	intHist	TOC34	signalling.G-	30.5;29.3.3	GTP binding		2	1	0.616387	37.904158	3547.438	3547.438	93.589672	50.488325	[02;]	[01;]	

* = click me

Column	description
J + K	count of replicates that showed a nonzero intensity
L	p value of testing intensity sums per protein
M-O	several distance measurements to sort with
P	maximal intensity amplitude per protein
R + S	Slices of peak intensity for WT and Mut
T ff.	mean intensities of replicates

- Column A: protein intensities over the slices with additional annotation information
- Column B: peptides associated with the protein
- Column C: peptides MaxQuant scores in relation to all other peptide scores
- Column D: protein intensity in relation to all other protein intensities

A

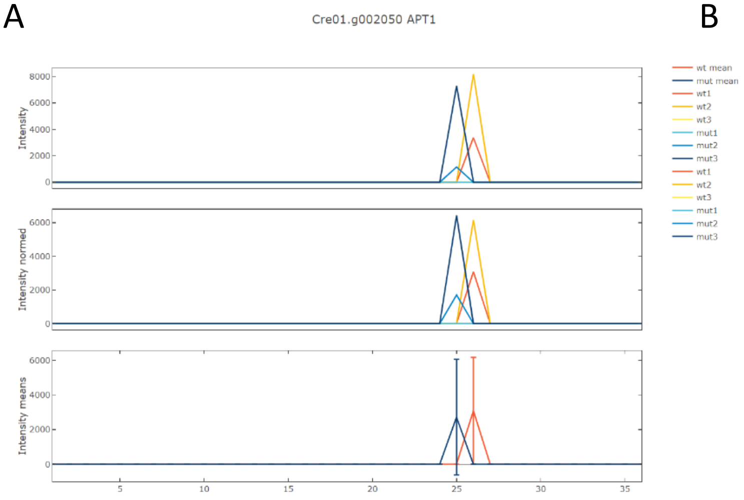

**Identifier**	Cre01.g002050
**Trivial name**	APT1
**Euclidean distance**	4107.81
**Average intensity sum (WT)**	85.7
**Average intensity sum (Mut)**	75.4
**Adjusted euclidean distance**	47.95
**MapMan annotation**	
**p-value** (Welch test) of intensity sums	0.89464

B

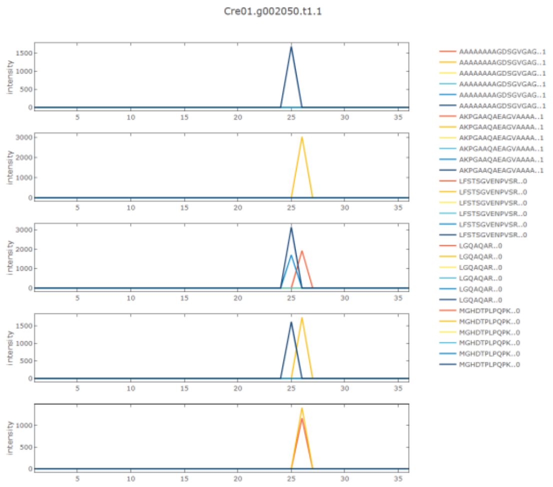

Cre01.g002050.t1.1

Trivial name: APT1
SeqCoverage [median]: 12.40
Mass: 77.32 kDa

Sequence	MissedCleavage	Mass [Da]	wtIntensity	muIntensity	Score
AAAAAAAAGDSGVGAGAEVEAKEQAK	1	2270.11	0.00	561.13	86.55
AKPGAAQAEAGVAAAAQEEVAK	1	2037.05	1006.77	0.00	66.05
LFSTSGVENPVSR	0	1391.70	641.25	1614.16	138.25
LGQAQAR	0	742.41	579.89	538.48	88.35
MGHDTPLPQPK	0	1219.60	855.87	0.00	64.82
			3083.8	2713.8	

C

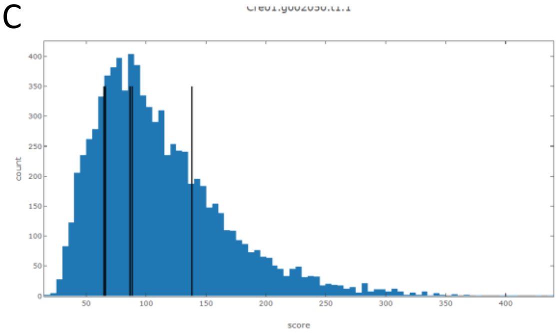

Cre01.g002050.t1.1

Trivial name: APT1
SeqCoverage [median]: 12.40
Mass: 77.32 kDa

Sequence	MissedCleavage	Mass [Da]	wtIntensity	muIntensity	Score
AAAAAAAAGDSGVGAGAEVEAKEQAK	1	2270.11	0.00	561.13	86.55
AKPGAAQAEAGVAAAAQEEVAK	1	2037.05	1006.77	0.00	66.05
LFSTSGVENPVSR	0	1391.70	641.25	1614.16	138.25
LGQAQAR	0	742.41	579.89	538.48	88.35
MGHDTPLPQPK	0	1219.60	855.87	0.00	64.82
			3083.8	2713.8	

D

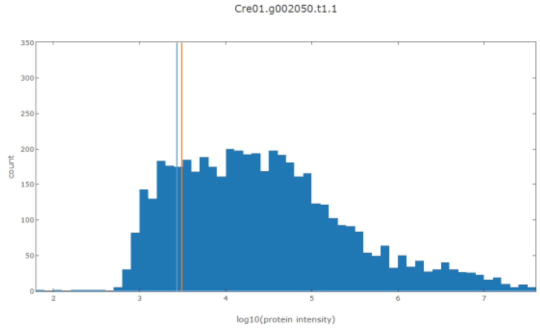

Cre01.g002050.t1.1

Trivial name: APT1
SeqCoverage [median]: 12.40
Mass: 77.32 kDa

Sequence	MissedCleavage	Mass [Da]	wtIntensity	muIntensity	Score
AAAAAAAAGDSGVGAGAEVEAKEQAK	1	2270.11	0.00	561.13	86.55
AKPGAAQAEAGVAAAAQEEVAK	1	2037.05	1006.77	0.00	66.05
LFSTSGVENPVSR	0	1391.70	641.25	1614.16	138.25
LGQAQAR	0	742.41	579.89	538.48	88.35
MGHDTPLPQPK	0	1219.60	855.87	0.00	64.82
			3083.8	2713.8	
